# A text-mining system for extracting metabolic reactions from full-text articles

**DOI:** 10.1186/1471-2105-13-172

**Published:** 2012-07-23

**Authors:** Jan Czarnecki, Irene Nobeli, Adrian M Smith, Adrian J Shepherd

**Affiliations:** 1 Department of Biological Sciences and Institute of Molecular and Structural Biology, Birkbeck, University of London, Malet Street, London, WC1E 7HX, UK; 2 Unilever R&D, Colworth Science Park, Sharnbrook, Bedfordshire, MK44 1LG, UK

## Abstract

**Background:**

Increasingly biological text mining research is focusing on the extraction of complex relationships relevant to the construction and curation of biological networks and pathways. However, one important category of pathway — metabolic pathways — has been largely neglected.

Here we present a relatively simple method for extracting metabolic reaction information from free text that scores different permutations of assigned entities (enzymes and metabolites) within a given sentence based on the presence and location of stemmed keywords. This method extends an approach that has proved effective in the context of the extraction of protein–protein interactions.

**Results:**

When evaluated on a set of manually-curated metabolic pathways using standard performance criteria, our method performs surprisingly well. Precision and recall rates are comparable to those previously achieved for the well-known protein-protein interaction extraction task.

**Conclusions:**

We conclude that automated metabolic pathway construction is more tractable than has often been assumed, and that (as in the case of protein–protein interaction extraction) relatively simple text-mining approaches can prove surprisingly effective. It is hoped that these results will provide an impetus to further research and act as a useful benchmark for judging the performance of more sophisticated methods that are yet to be developed.

## Background

### On the extraction of metabolic pathway information

An important goal of biological text mining is to extract relationships between named biological and/or medical entities. Until recently, the vast majority of research in this area has concentrated on extracting binary relationships between genes and/or proteins, most notably protein–protein interactions. However, attention is increasingly shifting towards more complex relationships, with a particular focus on biomolecular networks and pathways [1].

However, in spite of this new focus on networks and pathways, one of the most important sub-topics — the construction and curation of metabolic pathways — has largely been ignored. This is in contrast to the protein- and gene-centric focus of recent text-mining research: protein–protein interaction networks [2,3], signal transduction pathways [4-6], protein metabolism (synthesis, modification and degradation) [1], and regulatory networks [7,8]. This protein/gene-centric focus is also enshrined in the BioNLP’09 shared task on event extraction, an important initiative designed to galvanize community-wide effort to address the challenges of extracting information about complex events [1].

The only system that we are aware of that has an explicit focus on extracting metabolic pathway information from free text is the template-based EMPathIE [9], which is no longer under active development (R. Gaizauskas, personal communication). The aim of EMPathIE was to extract information about metabolic reactions together with relevant contextual information (including source organism and pathway name) from specific journals. When evaluated on a corpus of seven journal articles, EMPathIE achieved 23% recall and 43% precision [10].

Certain more generic systems may also be used for the same purpose, including the GeneWays system for “extracting, analyzing, visualizing and integrating molecular pathway data” [4], and the MedScan sentence parsing system [11], capable of extracting relationships between a range of biomedical entities including proteins and small molecules, and evaluated on a PPI extraction task by Daraselia *et al.*[12]. However, neither GeneWays nor MedScan are freely available and we are not aware of any published evaluation of their performance with metabolic pathway data.

It is interesting to note that the creators of GeneWays, in that system’s key publication, suggest signal-transduction pathways are an “easier target” for information extraction than metabolic pathways, and chose to evaluate its performance on the former rather than the latter [4]. Similarly Hoffmann *et al.* identify the extraction of metabolic information as a “special case” that has “specific problems” associated with it [13]. This perception may explain why relatively little attention has been paid to the task of extracting metabolic reactions from free text. The particular challenges that are characteristic of metabolic reaction extraction task include: 

• *Multiple entity types and entity mismatch*. Whereas protein–protein interaction networks, protein metabolism and signal-transduction pathways concern the entity-type *protein*, metabolic reactions involve both *enzymes* and *metabolites*. Moreover, there is a mismatch between the entities that most taggers address (*proteins*/*genes*, *small molecules*) and the entities that we wish to tag in metabolic pathways (*enzymes*, *metabolites*). Similar problems arise in the context of the extraction of protein–protein interactions owing to the fact that protein/gene taggers almost invariably fail to distinguish between proteins and genes. Only a subset of proteins are enzymes, and whereas the distinctive nomenclature associated with enzyme names may be beneficial to the extraction process (we address this point below), it has been argued that identifying the names of metabolites is more difficult than some other categories of chemical name [14].

• *Ternary (and n-ary) relationships*. Whereas the relationships in protein–protein interaction networks and signal-transduction pathways are typically binary (e.g. “protein A activates protein B”), metabolic relations are typically ternary (e.g. “enzyme C catalyzes the conversion of substrate D to product E”). Moreover, multiple substrates and/or products are commonplace, leading to further complexity. One consequence is that there is a greater potential for all the relevant entities in a metabolic reaction to be split over multiple sentences and for there to be a high incidence of anaphora usage.

One of the key themes of this paper is to address the question as to whether the extraction of metabolic reactions is, indeed, more difficult than the extraction of protein–protein interactions.

Although the fully-automated construction of networks and pathways from the literature may be the ultimate goal, a more practical focus for text mining systems in the immediate future is to provide assistance to database curators and model builders. Existing initiatives specifically designed to support database curation include PreBIND [15] and various tools [16] aligned with the task of curating FlyBase [17]. In this context, high recall is often deemed to be of paramount importance, although excessive numbers of false positives detract from the usability of such systems [18]. Existing initiatives designed to assist the curation of pathway and network databases include research that addresses the curation of Wnt signaling pathways [5] and an application designed to support the curation of chemical–gene–disease networks in the Comparative Toxicogenomics Database [19].

### A methodology for extracting metabolic reactions

Various approaches have been utilized for extracting relationships between biological entities described in free text, broadly ranging from simple methods based on the co-occurrence of terms to sophisticated natural language processing methods. Here we adopt an intermediate, rule-based and pattern-matching approach that combines lists of stemmed keywords with rules for rewarding and penalizing the occurrence of words depending on their location. Our approach can be viewed as an elaboration of several existing algorithms designed to extract protein–protein interactions (PPIs).

Indeed, the starting point for the algorithm developed here was the simple benchmark for PPI extraction presented in [20], which looks for ordered triplets of the form “protein name/interaction keyword/protein name”. The Co3 algorithm, available via the Whatizit suite of Web services [21], takes a similar approach, as does the algorithm devised by Ono *et al.*[22], but with the addition of simple parts-of-speech rules.

This kind of algorithm is easy to integrate with established named-entity recognition tools. Our algorithm builds on two state-of-the-art named-entity taggers: BANNER [23] for recognizing gene/protein names; and OSCAR3 [24,25] for identifying the names of chemical entities.

However, one important difference in the algorithm we have developed arises from the intrinsic complexity of the relationships we are seeking to extract. Different permutations of assigned entities within a given sentence are scored separately, although there are rules to ensure that implausible permutations are ignored. Details are given in the Methods section below.

In terms of performance, we might anticipate that our algorithm will give higher precision, but lower coverage, than simple co-occurrence methods; and higher coverage, but lower precision, than NLP-based methods. It is interesting to note that the simple algorithm used in [20] proved remarkably effective when evaluated against some well-regarded NLP-based approaches. When other factors are taken into account, such as execution speed and ease of installation, simple algorithms of this type are worthy of serious attention.

### A metabolic reaction extraction task

When considering how to evaluate our system, we found that existing corpora — even those with many sentences that contain the names of at least two small molecules, for example GENIA [26] and the metabolite corpus developed by Nobata and coworkers [14] — do not contain significant amounts of metabolic information relevant to our chosen target. Given that we perceive support for metabolic pathway curation as the ultimate goal of our research, we chose to assess how many reactions belonging to a given metabolic pathway our system is able to extract from papers known to be relevant to that pathway. To this end, three contrasting pathways were chosen from the EcoCyc database [27].

Our approach to evaluation differs in two additional respects from the recent protein-centric BioNLP shared task on complex relationship extraction [1]: rather than abstracts alone, we use additional sections of full text articles (see below); and we do not use pre-annotated entities. For the shared task, gold-standard annotations of protein names were provided from the outset. It was argued at the time that this would not have a major impact on the results, whilst it was acknowledged that it detracted somewhat from the task’s “realism” [1]. However, an analysis by Kabiljo and coworkers demonstrated that the use of putative entity names that have been predicted using entity taggers (with an associated error rate of around 15%) instead of true, gold-standard entity names (extracted manually from the literature) can have a surprisingly large impact on relationship extraction scores, with “a fall of around 20 percentage points [in F-score] being commonplace” [20].

Although our approach to evaluation has been previously adopted elsewhere (see, for example, Rodríguez-Penagos *et al.*[8]), we acknowledge that it is “unrealistic” in that all papers are known to be relevant in advance. Although this is an important caveat, we believe the identification of relevant papers (e.g. with respect to the species of interest) is the task of a separate information retrieval component and that our evaluation of our system’s ability to extract metabolic reactions is highly informative.

## Methods

### Existing text-mining resources

OSCAR3 [28] is an open source tool for identifying chemistry-specific terms in free text that uses an approach that combines n-grams, regular expressions and heuristic rules with access to a chemical dictionary [25]. There are relatively few competitor taggers that annotate the names of chemical entities, and OSCAR3 is arguably the most mature and widely-used of those that are freely available. It performed exceedingly well in a recent independent evaluation by Wiegers *et al.*[19], retrieving well over 90% of curated chemical “actors”, and had high recall rates (over 85%) when applied to a new manually-annotated test metabolite corpus [14].

Although OSCAR3 is designed to tag enzymes as well as small molecules, its strategy for identifying enzyme names is limited to checking for single words with the suffix “-ase” or “-ases”. However, we found this to be inadequate in practice; in a preliminary examination of the context in which enzymes are mentioned in our training set of sentences, we discovered a significant number of cases in which the standard nomenclature was not used. For instance, consider the following sentence fragments (in which we have italicized the enzyme entity): 

“The *protein encoded by the hisF gene* has an ammonia-dependent activity”

“The *hisB gene product* appears to be a bifunctional enzyme”

Moreover, even when the name of the enzyme ends in “-ase”, it often comprises multiple words, only one of which is tagged by OSCAR3. Hence, in the sentence:

“Therefore, cleavage of acetoin might occur by a reaction that is analogous to the oxidative-thiolytic cleavage of pyruvate to acetyl-CoA and CO2 catalyzed by the pyruvate dehydrogenase multienzyme complex.”

we would like to annotate the full name *pyruvate dehydrogenase*, or perhaps *pyruvate dehydrogenase multienzyme complex*, rather than just the single word *dehydrogenase* that OSCAR3 annotates. It is also worth noting that OSCAR3’s mark-up nomenclature for enzymes (*type=“CM”*) is the same as that for chemical entities in general. For these reasons we decided to use a generic gene/protein name tagger in our system to identify the names of putative enzymes, rather than OSCAR3.

Nevertheless, most enzyme names do end in “-ase” or “-ases”, and there are potential benefits to taking these distinctive suffixes into account when choosing between multiple putative enzyme names (see below).

There are several free gene/protein name taggers available. We chose BANNER (v02) [23,29], an open source tool that applies a conditional random fields-based approach to a range of orthographic, morphological and syntactic features, on the grounds that it was the best performing tagger in our earlier, multi-corpus evaluations [20].

Sentence splitting and tokenization were carried out using the Apache OpenNLP toolkit v1.4.3 [30] with biochemical models available from the Jena University Language & Information Engineering (JULIE) lab [31]. All these resources are open source.

### Reaction extraction algorithm

Given text in which the names of putative proteins and small molecules have been tagged, our algorithm proceeds in three key stages: a sentence selection phase; an entity assignment phase; and an assignment scoring phase.

#### Sentence selection

The algorithm begins by selecting sentences containing at least two small molecules. Our working assumption is that sentences of interest will contain the names of both a substrate and a product, but not necessarily the name of an enzyme; it is sometimes possible to correctly identify substrate and product even when the name of an enzyme is not found (e.g. when it is mentioned in a separate sentence). In our training corpus (described below), 30% of the sentences that describe a metabolic reaction (and selected without reference to whether the name of an enzyme is present or not) do not contain the name of an enzyme.

#### Entity assignment

Given a selected sentence, most (but not all) potential orderings of putative enzyme(s), substrate(s) and product(s) occurring within the sentence are then considered in turn, or — in the absence of a putative enzyme name — orderings of substrate(s) and product(s). The possibility that the reaction has multiple substrates and/or products is taken into account during this scoring phase. Consider, for example, the sentence: 

“L-Arabinose isomerase catalyzes the conversion of L-arabinose to L-ribulose, the first step in the utilization of n-arabinose by *Escherichia coli B/r*.”

Here BANNER tags *L-Arabinose isomerase* as a putative protein, and OSCAR tags *L-arabinose*, *L-ribulose* and *n-arabinose* as putative small molecules. Ten different ways that the entities enzyme, substrate and product may be assigned to the tagged names are deemed suitable for consideration during the scoring phase. These assignments are given in Table 1.

**Table 1:** Assignments of the entities enzyme (E), substrate (S) and product (P) for a sample sentence

**L-Arabinose isomerase**	**L-arabinose**	**L-ribulose**	**n-arabinose**
E	S	P	P
E	S	S	P
E	P	S	S
E	P	P	S
E	S	P	
E	S		P
E		S	P
E	P	S	
E	P		S
E		P	S

The ten assignments of E, S and P for the sentence “L-Arabinose isomerase catalyzes the conversion of L-arabinose to L-ribulose, the first step in the utilization of n-arabinose by *Escherichia coli B/r*”. Given that L-Arabinose isomerase is the only tagged protein, it is deemed to be the enzyme in all cases, whereas different numbers and orderings of substrates and products are possible, given the presence of three tagged small molecules (L-arabinose, L-ribulose and n-arabinose). Note that other potential orderings (namely E-P-S-P and E-S-P-S) are not considered, as they are deemed highly unlikely to occur in practice.

#### Assignment scoring

Given a sentence to which the entities substrate, product and (optionally) enzyme have been assigned, each assignment is then awarded a separate score based on the following criteria: 

• Each word occurring between the first and last assigned substrate and product — the entities *L-arabinose* and *n-arabinose* in the exemplar sentence above — and that does not belong to the name of any additionally-assigned entities — *L-ribulose* in this exemplar sentence — incurs a small penalty (-0.1 points per word).

• If a keyword is found at an appropriate location relative to one or more entities, the assignment is awarded a positive score (+2 points per keyword).

• If a keyword is found in an inappropriate location, a penalty (of -1 point) is incurred.

• A bonus (of +2 points) is awarded when both a reaction and production keyword are found, provided they are in appropriate locations.

Keywords fall into the following categories: reaction word stems (e.g. *add*, *convert*, *hydrolys*, *dimeris*); production word stems (e.g. *form*, *give*, *produc*, *synthesi*); variants of the verb *catalyze*; prepositions (e.g. *to*, *from*, *by*); and the coordinating conjunction *and*. Stemming was performed using a Java implementation [32] of the standard Porter stemming algorithm [33]. Scoring locations include: between an assigned enzyme and substrate for reaction keywords; between a substrate and product for reaction keywords, for production keywords and for the prepositions *to* and *into*; and between the last two assigned products/substrates for the word *and*. An example of an inappropriate location for a production keyword is before an assigned substrate.

As an example, here is the scoring for our exemplar sentence given the following assignments: 

Enzyme = *L-Arabinose isomerase*

Substrate = *L-arabinose*

Products = *L-ribulose* and *n-arabinose*

• Reaction keyword *conversion* found between the enzyme and substrates: +2 points.

• Preposition *to* found between substrate and products: +2 points.

• Both a reaction word and a production word have been found: +2 points.

• Word *catalyzes* found: +2 points.

• Penalty for words between first and last entities: -0.8 points.

This gives a total score of +7.2 points.

The list of assignments is then ranked by score and the final predictions are made from this list, highest score first. Multiple assignments may be used to make predictions, thereby enabling the algorithm to identify multiple reactions within a single sentence.

The keyword lists and weightings used in the algorithm were chosen as follows: 

• The reaction keyword list was assembled manually with specific reference to the nomenclature used in the Enzyme Commission classification [34].

• The production keyword list, together with the set of prepositions and conjunctions, were assembled manually from an examination of the literature, from our own knowledge of the field, and using a thesaurus.

• The weightings (bonuses and penalties) used for each component when generating a score for a given assignment were derived from a small training corpus described below.

It is worth noting that an attempt to automatically compile a keyword list from verbs found between a tagged protein entity and a tagged small molecule in the GENIA corpus (a process analogous to that carried out by Kabiljo *et al.* in the context of PPI extraction [20]) proved insufficiently discriminatory to be useful, as the false positive rate was too high.

The program used to generate the metabolic reaction extraction results presented in this paper is available in Additional file 1 and an explanatory worked example is given in Additional file 2.

### Training and evaluation

#### Training corpus

A small training corpus was used to set the weighting for the various scoring rules described in the previous section. This corpus consists of sentences containing the names of at least two small molecules selected manually from the literature referenced in the EcoCyc database [27] for various metabolic pathways, but excluding the specific pathways used subsequently for evaluation; 100 sentences were manually selected that describe at least one reaction each (with at least one named substrate and one named product), together with 100 sentences containing the names of multiple small molecules, but that do not describe a specific reaction. It is important to note that these were the only criteria used to select sentences from the set of referenced papers. No attempt was made to exclude “difficult” sentences, hence the corpus contains the following complex sentence with multiple reactions: 

“ZEP catalyses the epoxidation of zeaXanthin to produce epoxycarotenoid; NCED catalyses the cleavage reaction of epoxycarotenoids to produce xanthoxin (the first C15 intermediate); and AAO catalyses the final step of ABA biosynthesis, which converts ABA aldehyde to ABA.”

Half of the sentences (i.e. 50 describing interactions, 50 describing no interactions) were used to manually adjust the weightings of the various scoring components described above in order to find a good combination for differentiating between true positives (i.e. true interactions with entities correctly assigned) and false positives (i.e. non-interactions, or interactions with entities misassigned). In addition, we chose a scoring threshold (set to 3.0); any permutation of entity assignments that gives a score below the threshold is eliminated from further consideration. The effectiveness of the chosen weightings was evaluated using the remaining set of 100 sentences. No attempt was made to highly optimize the choice of weightings and thresholds, as our sample size of sentences was relatively small and unlikely to be highly representative of relevant literature as a whole.

#### Evaluation pathways

Rather than create a set of manually-annotated sentences or abstracts to evaluate our method, we decided to assess performance against manually-curated pathways in the EcoCyc database. This is a similar approach to that adopted by Yuryev *et al.*[6] in the context of automated signaling pathway construction and Rodríguez-Penagos *et al.*[8] when evaluating the automated reconstruction of a bacterial regulatory network.

**Figure 1:**
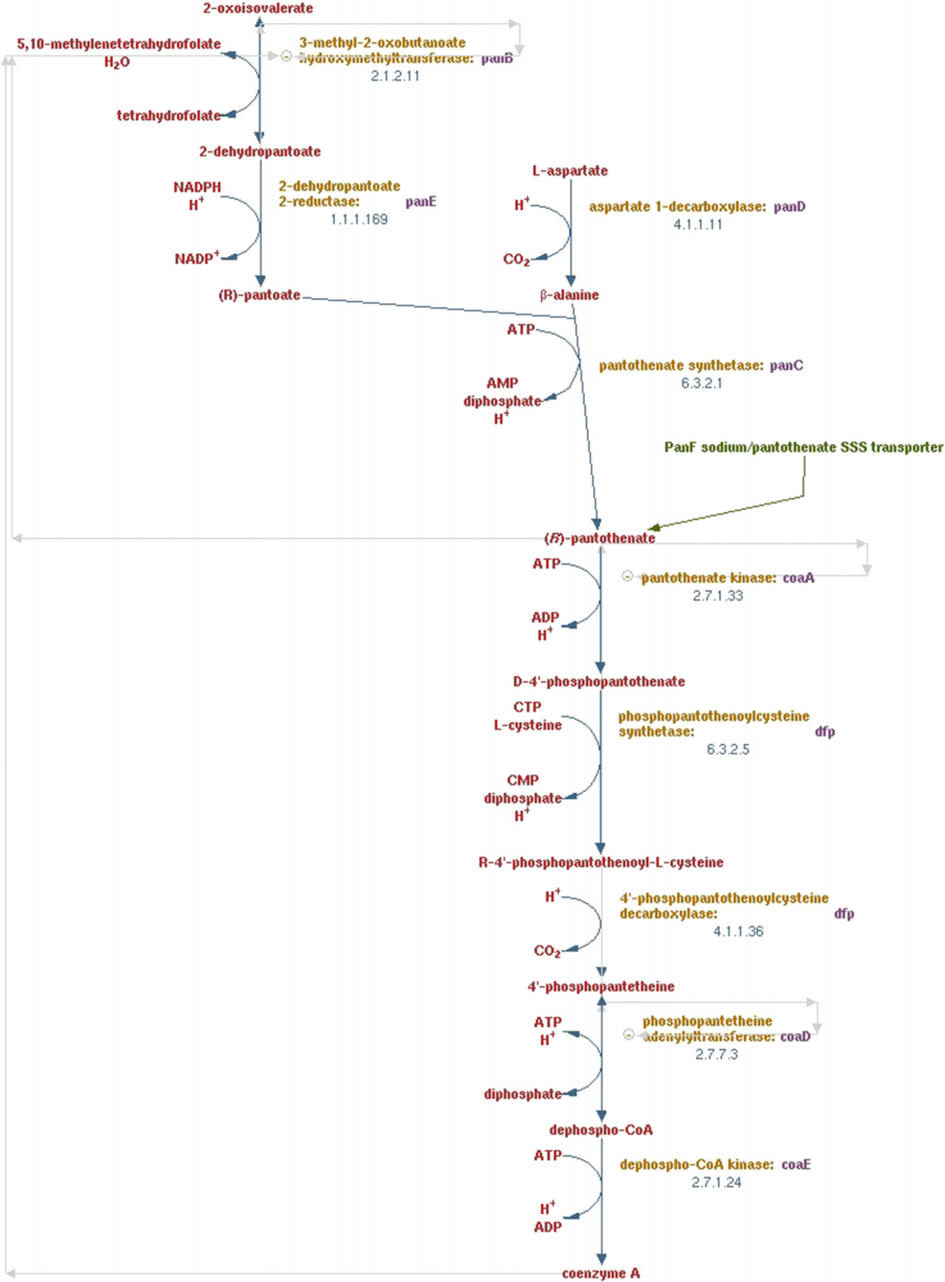
**The pantothenate and coenzyme a biosynthesis pathway.** A diagram of the pathway obtained using the BioCyc pathway viewer [35].

We chose three pathways from EcoCyc and collected the original papers cited in each of these EcoCyc entries: the pantothenate and coenzyme A biosynthesis pathway (8 papers), shown in Figure 1; and the tetrahydrofolate biosynthesis pathway (13 papers) and the aerobic fatty acid *β*-oxidation I pathway (11 papers), shown in Additional file 2. All three pathways are from *E. coli K-12 substr. MG1655*. All reactions in all three pathways have at least one substrate, product and enzyme; some reactions have multiple substrates and/or products, but there is never more than one enzyme.

We chose to annotate only the Abstract and Introduction of the referenced papers using our metabolic reaction system and compare the results to the relevant pathways within EcoCyc. Our decision to exclude the Methods, Results and discussion sections was in part a pragmatic one (it reduced the amount of text we needed to examine manually in order to evaluate the performance of our system), but was also guided by previous research concerning the information content of the different sections of full-text articles. For example, Shah *et al.*[36] undertook an analysis of the distribution of protein and gene names in 104 articles, and concluded that the Abstract and Introduction were the best sources of information about entities and their interactions, with the Methods and, to a lesser extent, the Results sections often proving problematic (for example, keywords unique to the Methods section commonly refer to reagents and experimental techniques).

We also carried out a complementary analysis against a collection of short passages of text containing known reactions which we obtained from the Reactome database [37]. Using the database we were able to link together metabolic reactions and the short passages of text describing them. Our intention was to automate a comparison between the gold-standard reactions in Reactome and the reactions extracted by our method. This required that all entities in the reaction must be present in the describing text and the entities must all be able to be trivially matched with their corresponding mentions with the text. This filtering produced 193 suitable passages of text with corresponding metabolic reactions.

#### Measuring performance

To gain a rounded picture of how well our system performs, we considered the quality of its predictions for different aspects of our evaluation data: the entities (enzymes, small molecules) within a pathway; the metabolic reactions within a pathway; the binary relationships (enzyme-substrate, enzyme-product, substrate-product) within a reaction; and whole pathways.

Given that primarily we compared our predictions to manually-curated pathways, rather than to gold-standard corpus annotations, we chose to adopt a similar approach to measuring performance to that of Rodríguez-Penagos *et al.* (2007) [8]. However, in our preliminary evaluation of entity tagger performance, we used gold-standard manually-annotated corpora, rather than curated pathways. In this context we were able to calculate the standard recall, precision and F-score metrics used in the majority of text mining research. Consequently the main performance measures we used are: 

• *Recall(C)*: Of the reactions/relationships/entities within a corpus of texts, the percentage that have been extracted — here ’C’ stands for ‘corpus’.

• *Recall(P)*: Of the reactions/relationships/entities within a manually-annotated pathway, the percentage that have been extracted — here ’P’ stands for ‘pathway’.

• *Precision*: Of the extracted reactions/relationships/entities, the percentage that are correct.

• *F-score*: The weighted harmonic mean of recall(C) and precision.

Note that, in evaluating recall, we only take into account the primary metabolites belonging to the main route along the metabolic pathway. Hence side metabolites, such as *ATP *→* ADP* + *P*_*i*_, are ignored. We took this approach because it is common practice for authors to omit details about side metabolites from published papers, leaving them to be inferred by the reader.

When judging the accuracy of named entity taggers, there is a choice to be made between “strict” matching criteria (where the tagger is required to match a given name exactly) and “sloppy” matching criteria (where the tagger is not required to match the name boundaries exactly to score a “hit”). For example, consider the following tagged sentence fragment: 

“…is a key precursor of the <**molecule** > 4^*′*^-phosphopantetheine </**molecule** > moiety of…”

Using sloppy matching criteria, credit is given for annotating *phosphopantetheine*, *4*^*′*^*-phosphopantetheine* or *4*^*′*^*-phosphopantetheine moiety*, but also for *key precursor of the 4*^*′*^; whereas strict matching criteria require an exact match to *4*^*′*^*-phosphopantetheine*.

In this research we adopted sloppy matching criteria on the grounds that they have proved more informative than strict criteria in the context of gene/protein named-entity recognition in general, and of gene/protein relationship extraction in particular. With respect to named-entity recognition, in the vast majority of cases where a match was found using sloppy criteria but not with strict criteria, the core part of the entity name was correctly identified [38]. Strict criteria were deemed misleading because they are highly sensitive to the essentially arbitrary choices made when drawing up annotation guidelines for the evaluation corpora — for example, whether the word “mouse” is part of the protein name in the phrase “mouse oxytocin”. With respect to named-entity recognition in the specific context of relationship extraction, a manually corrected F-score was only 4 percentage points lower than the sloppy F-score, but 20 points greater than the strict F-score [20].

In the data sets used for this research there are a few examples where sloppy matching criteria arguably give a misleading impression about how well a complex entity name has been tagged. With sloppy matching, both the following examples of sub-optimal tagging score a “hit”: 

• The significant truncation of the long entity name *Geranyl pyrophosphate:(-)-endo-fenchol cyclase* to *endo-fenchol cyclase*;

• The splitting of single entity *TPS-d3 family members of conifer diterpene synthases* into the two tagged entities *TPS-d3* and *conifer diterpene synthases*.

However, such examples are comparatively rare, and we have concluded that the number of false negatives that appear to be true negatives with strict criteria is a more significant problem than the number of false positives that appear to be true positives with sloppy criteria.

## Results and discussion

### Pre-evaluation of entity taggers

We performed a preliminary evaluation of the performance of BANNER and OSCAR3 on the GENIA corpus [39], which contains 2,000 biomedical abstracts related to the specific topic of human blood cell transcription factors. GENIA was chosen because it contains annotations for a broad range of biological and chemical entities. We additionally tested OSCAR3 using the dedicated Fraunhofer SCAI chemical corpus [40], which contains 101 abstracts from chemistry papers. Neither tool was developed using either of these corpora: BANNER was trained on the BioCreAtIvE corpus [41], and OSCAR3 combines knowledge-based heuristics with the chemical dictionary ChEBI [42].

BANNER scored 72% for precision, recall(C) and F-score on GENIA. This is roughly in line with our expectations; it has been previously shown that a range of protein/gene name taggers perform less well on GENIA than on some other widely-used corpora, and that this is (at least in part) attributable to the chosen annotation criteria (see, for example, the analysis in [43]).

**Figure 2:**
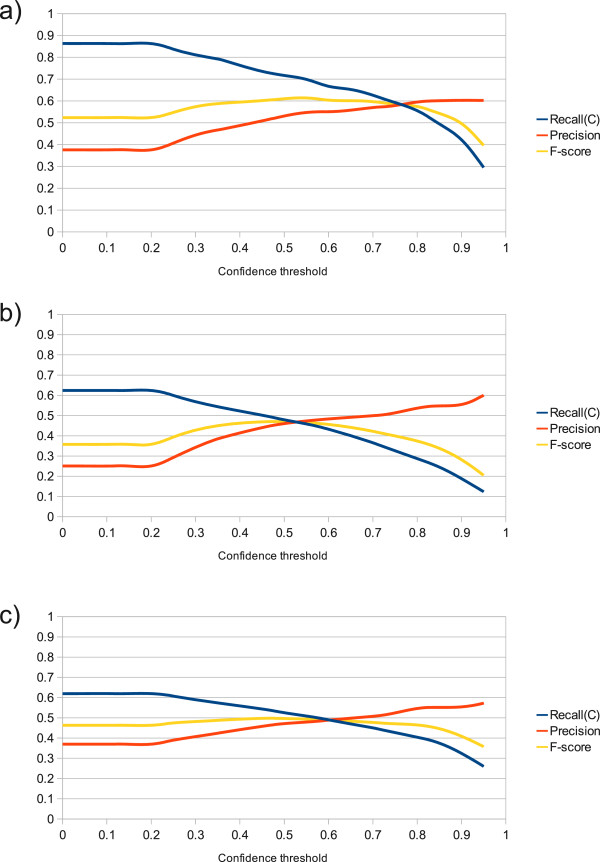
**Graphs showing the performance of OSCAR3 at a range of confidence thresholds.** Performance is shown under the following conditions: **a)** when applied to the SCAI chemical corpus; **b)** when applied to the GENIA corpus without acronym detection; and **c)** when applied to the GENIA corpus with acronym detection. The y-axis gives the recall(C), precision and F-score values in the range 0 to 1.

The results for OSCAR3 are more interesting and are presented in Figure 2. Two features stand out from these results: the best performance of OSCAR3 on both corpora is worse than we had expected from results presented elsewhere [19], with peak F-scores of 62% and 48% on the Fraunhofer SCAI corpus (Figure 2a) and the GENIA corpus (Figure 2b) respectively; and the performance on the GENIA corpus is significantly worse than that on Fraunhofer SCAI.

A preliminary examination of the tagged text generated by OSCAR3 for both corpora indicated that a significant proportion of the false positives were attributable to acronyms being tagged as the names of chemicals. This is a known problem (identified in the original OSCAR3 paper by Corbett & Murray-Rust [24]) and one that the authors advocate addressing at the level of the wider text-mining framework.

In this spirit, we developed a simple method for resolving acronyms. Any putative acronym (i.e. any uppercase token of more than one letter) is deemed to be a false positive unless either a) a defining chemical name is found in the text preceding it, or b) OSCAR3 gives it a confidence score of 0.5 or more. The latter criterion is used to allow for the presence of commonly occurring molecules for which acronyms are frequently used without explicit definition (e.g. NAD). This approach achieved a significant improvement in precision at the cost of a negligible drop in recall (Figure 2c). Bearing these results in mind, we henceforth used OSCAR3 with the threshold set to zero, thereby maximizing recall.

We also used our training corpus of sentences containing the names of at least two small molecules (see above) to assess whether, in cases where BANNER tags multiple protein names within a single sentence, it is advantageous to prefer names that end in “-ase” or “-ases”. Of the 77 enzyme names in the training corpus, 60 end in “-ase(s)”. As expected, the suffix “-ases” commonly occurs when a text refers to a class of enzymes in general, whereas the suffix “-ase” is used when a specific enzyme is being discussed in the context of a particular reaction.

Where BANNER tags multiple names within a single sentence, we concluded that giving preference to names that have the ending “-ase(s)” is potentially beneficial in the vast majority of cases. This is mainly attributable to the tendency of BANNER to tag multiple terms that refer to the same entity; by giving precedence to a term that ends in “-ase(s)”, the typical effect is to select the full name of the enzyme ahead of its tagged abbreviation and/or EC number. For simplicity, we have not incorporated this approach in our current method. However, a small increase in the score assigned to putative enzymes that have names ending in “-ase(s)” is, we believe, worth further consideration.

### Performance of entity taggers on metabolic corpora

We began by undertaking a standard analysis of tagger performance by evaluating their scores for all the entities in the Abstract and Introduction of each of the papers associated with our three evaluation pathways. Results are shown in Table 2.

**Table 2:** The tagging performance of BANNER and OSCAR3

	**Protein names tagged**	**Small molecule names**
	**by BANNER**	**tagged by OSCAR3**
* Pantothenate and coenzyme A biosynthesis pathway*		
Recall(C) (%)	81 (112/139)	96 (329/343)
Precision (%)	85 (112/132)	86 (329/384)
F-score (%)	83	91
* Tetrahydrofolate biosynthesis pathway*		
Recall(C) (%)	93 (250/268)	82 (528/647)
Precision (%)	76 (250/327)	95 (528/558)
F-score (%)	84	88
* Aerobic fatty acid β-oxidation I pathway*		
Recall(C) (%)	91 (341/376)	81 (456/565)
Precision (%)	82 (341/414)	92 (456/494)
F-score (%)	86	86

The tagging performance of the NER tools when applied to the Abstracts and Introductions from papers referenced in EcoCyc with respect to our three evaluation pathways. Taking the BANNER column for the pantothenate and coenzyme A biosynthesis pathway as an example, the numbers in brackets indicate that BANNER correctly identified 112 out of the 139 protein names (recall row); and of the 132 names it tagged, 112 were correct (precision row). The OSCAR3 results are with a confidence threshold of zero.

Performance here is significantly higher than it was for GENIA. It is worth noting that, in the case of BANNER, the performance on this corpus is very similar to its performance on gene/protein interaction corpora such as AIMed [44] and the LLL training corpus created for the 2005 LLL challenge [45], for which F-scores of 82.9% and 84.1% were reported in [20].

### Relationship extraction

**Figure 3:**
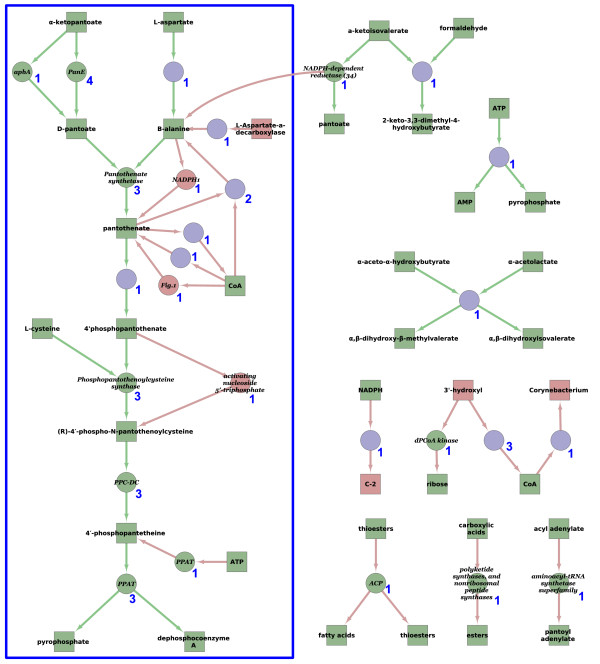
**A network showing the reactions predicted from the eight source papers for the pantothenate and coenzyme A biosynthesis pathway.** Squares are small molecules, circles are enzymes, and a pair of arrows is used to denote a single reaction (the first for the interaction substrate-enzyme, and the second for the interaction enzyme-product). Items labeled green are correct; items labeled red are incorrect. The number next to a reaction indicates the number of times that reaction was extracted from the set of source texts. The reactions on the right-hand side of the figure (lying outside the blue rectangle) are reactions extracted by our algorithm that are not part of the manually-annotated pantothenate and coenzyme A biosynthesis pathway from EcoCyc given in Figure 1.

Our metabolic reaction extraction results (with and without the correct assignment of enzymes taken into account) for all three evaluation pathways, are shown in Table 3. The same results broken down into binary interactions (substrate–product, substrate–enzyme and product–enzyme), along with the results for the Reactome dataset, are shown in Table 4. Note that the number of binary pairs is larger than the number of reactions, because some reactions comprise multiple substrates and/or products. A visual summary of the complete set of results for the smallest of the three pathways (the pantothenate and coenzyme A biosynthesis pathway) is given in Figure 3. Equivalent figures for the tetrahydrofolate biosynthesis and the aerobic fatty acid *β*-oxidation I pathways are given in Additional file 2, together with a set of example sentences annotated with the putative entities and relationships extracted by our system.

**Table 3:** The performance of our metabolic reaction extraction method on three evaluation pathways

	**Correct reactions**	**Correct**
	**(ignoring enzyme)**	**(including enzyme)**
* Pantothenate and coenzyme A biosynthesis pathway*		
Recall(P) (%)	78 (7/9)	56 (5/9)
Precision (%)	59 (24/41)	41 (17/41)
* Tetrahydrofolate biosynthesis pathway*		
Recall(P) (%)	90 (9/10)	70 (7/10)
Precision (%)	60 (39/65)	38 (25/65)
* Aerobic fatty acid β-oxidation I pathway*		
Recall(P) (%)	29 (2/7)	29 (2/7)
Precision (%)	30 (11/37)	14 (5/37)

Taking the “correct reactions (ignoring enzymes)” column for the pantothenate and coenzyme A biosynthesis pathway as an example, the numbers in brackets indicate that our algorithm correctly identified 7 out of the 9 reactions in the curated EcoCyc pathway (recall row), giving 78%; and of the 41 identified interactions (precision row), 24 were valid reactions (irrespective of whether they belong to the pathway or not), giving 68%. A reaction for which the susbtrate(s) and product(s) have been correctly assigned, but not the enzyme, is deemed correct in column two, but incorrect in column three.

**Table 4:** Binary interaction extraction for all three evaluation pathways

	Substrate–product	Substrate–enzyme	Product–enzyme	Total
* Pantothenate and coenzyme A biosynthesis pathway*				
Recall(P) (%)	67 (10/15)	58 (7/12)	55 (6/11)	61 (23/38)
Precision (%)	59 (35/59)	65 (13/20)	59 (13/22)	60 (61/101)
* Tetrahydrofolate biosynthesis pathway*				
Recall(P) (%)	82 (9/11)	64 (7/11)	70 (7/10)	78 (25/32)
Precision (%)	48 (55/114)	62 (28/45)	58 (26/45)	53 (109/204)
* Aerobic fatty acid β-oxidation I pathway*				
Recall(P) (%)	20 (2/10)	38 (3/8)	38 (3/8)	31 (8/26)
Precision (%)	40 (12/30)	80 (8/10)	67 (6/9)	53 (26/49)
* Reactome dataset*				
Recall(P) (%)	63 (737/1167)	37 (166/439)	36 (157/439)	52 (1060/2045)
Precision (%)	88 (749/856)	72 (169/235)	67 (157/234)	81 (1075/1325)

Numbers in brackets were calculated as for Table 3.

Fair and meaningful comparisons within the field of biological text mining are extremely difficult; for example, a single system may give a wide range of different performances even when applied to different corpora within the same sub-domain. In this research, a prominent feature of the results (as presented in Tables 3 and 4) is that our algorithm performs noticeably less well on the aerobic fatty acid *β*-oxidation I pathway than on the other two pathways. To a significant extent this appears to be attributable to the distinctive ways that reactions in fatty acid pathways are commonly described, for example in terms of molecular addition (with no explicit product mentioned): 

"Enoyl-CoA hydratase catalyzes the second reaction of the fatty acid β-oxidation, i.e., the syn addition of water to α,β-unsaturated fatty acyl-CoA thioesters."

However, in the absence of a substantially larger data set, we are unable to draw firm conclusions.

Notwithstanding these caveats and challenges, we note (with considerable caution) that our results appear to be somewhat better than those achieved using the EMPathIE system. However, no direct comparison is possible.

Of the 182 reactions extracted from the Reactome dataset, 53 were perfect extractions. A manual analysis on extracted reactions that matched poorly with the gold-standard reactions showed that 41 of the reactions in the dataset where described in a form unlikely to be used in a journal article. Consider, for example, the following passage: 

"At the beginning of this reaction, 1 molecule of ’Oxygen’, 1 molecule of ’H2O’, and 1 molecule of ’5-Hydroxytryptamine’ are present. At the end of this reaction, 1 molecule of ’NH3’, 1 molecule of ’5-Hydroxyindoleacetaldehyde’, and 1 molecule of ’H2O2’ are present."

We should, of course, be even more cautious when making comparisons between different sub-domains and where the evaluation strategies are different. Nevertheless, we think it is useful to consider how the performance of our method for extracting metabolic reactions compares to that in the well-studied sub-domain of gene/protein interaction extraction. Here (in Table 5) we briefly compare the performance of our method with the reported performance of three contrasting gene/protein interaction tools: the rule-based RelEx method [46], which was the best-performing method in the evaluation reported in [20]; the NLP tool AkanePPI [47] trained on the BioInfer corpus [48]; and the simple baseline(k) algorithm in [20].

**Table 5:** Performance comparison of gene/protein extraction tools with our metabolic reaction extraction method

			
			
Method	Interaction type	Precision	Recall
RelEx	Protein-protein	39-80	45-72
Baseline(k)	Protein-protein	23-54	52-67
AkanePPI			
(trained on BioInfer)	Protein-protein	29-77	40-56
Method described			
in this paper	Substrate-product	40-88	20-82
Method described			
in this paper	Substrate-enzyme	62-80	37-64
Method described			
in this paper	Product-enzyme	58-67	36-70

The range of scores for the gene/protein extraction tools are for five corpora as evaluated in [20]. The scores for our metabolic reaction extraction method summarize those in Table 4, i.e. they are broken down into the same three binary interactions and the range is for the three evaluation corpora and the Reactome dataset.

These results suggest both that our method performs reasonably well when placed in the wider context of biomedical relationship extraction, and that metabolic reaction extraction is more tractable than has hitherto been assumed.

There are a number of ways that the method could be improved, for example by incorporating techniques for handling negation (and speculation) and resolving anaphora, and the system might benefit from using more sophisticated tools in place of our present simple strategies, such as the widely-used Acronym Resolving General Heuristic (ARGH) program [49]. More generally, we should anticipate that more sophisticated NLP approaches will give better precision at the potential cost of lost recall and greater complexity. But perhaps more interesting is the fact that our relatively simple method performs so well, especially in light of prior assumptions that this is a particularly challenging sub-domain. There are several reasons why this may be the case: 

• Whole reactions are commonly described in a single sentence.

• A single sentence commonly describes a single reaction and nothing else.

• Entity taggers appear to be reasonably accurate in a metabolic context, with most enzyme names having the ending “-ase” or “-ases”.

• Keyword lists appear reasonably discriminatory when distinguishing metabolites from non-metabolites and substrates from products.

• Most reactions are described multiple times in the literature; typically at least one occurrence will be worded in such a way that the information is relatively easy to extract.

## Conclusions

In this paper we have presented a simple method for extracting metabolic reactions from free text. We have shown that it successfully extracted a high percentage of reactions for two out of three pathways; the third pathway, dealing with fatty acid metabolism, proved particularly challenging owing to the distinctive way in which reactions are described (for example, in terms of molecular addition). In so far as comparisons with broadly comparable methods are possible, it appears that our approach performs rather well; that, at least, is what our brief comparison with the performance of gene/protein interaction extraction methods suggests, with both precision and recall at comparable levels.

Given that information about secondary metabolites such as ATP is frequently omitted from source papers, we have focussed on the extraction of primary metabolites, rather than side metabolites, in the evaluations we present here. Clearly, this lack of information about side metabolites in the literature is an obstacle to the fully automated construction of complete metabolic pathways using text-mining methods. However, a more realistic goal for a metabolic text mining system is to support manual curation. In this latter context, we believe our evaluations show that our method could prove immediately useful to database curators, who are already used to having to infer the side metabolites when metabolic reactions are incompletely specified in the literature.

Ultimately, we believe there is one overriding implication of this research: that the extraction of metabolic reactions may be more tractable than previously assumed and therefore worthy of more widespread attention within the biological text-mining community in the immediate future.

## Competing interests

The authors declare that they have no competing interests.

## Author’s contributions

JC wrote the programs used in the experiment and analysed the results. AJS participated fully in the experimental design and analysis of the results and drafted the manuscript. IN and AMS contributed to the experimental design. All four authors co-edited, read and approved the final manuscript.

## Supplementary Material

Additional file 1 **ReactionExtractor.** An example Java program, provided as a runnable .jar file, implementing the algorithm described in the paper. The program takes a plain text file as input and outputs all predicted reactions from the input text. Running instructions are included in the archive. The open source tools described in the paper (BANNER, OSCAR3 and OpenNLP) are all included in the archive. Click here for file

Additional file 2 **SupplementaryMaterial.** An archive containing a detailed, worked example of the algorithm and the reconstructions of the tetrahydrofolate biosynthesis pathway and the fatty acid *β*-oxidation I pathway, together with a set of example sentences annotated with the putative entities and relationships extracted by our system. Click here for file
