# Supplementary material for: A text-mining system for extracting metabolic reactions from full-text articles
Source: BMC Bioinformatics. 2012 Jul 23;13:172. doi: 10.1186/1471-2105-13-172 (PMC3475109; doi:10.1186/1471-2105-13-172)
Supplement: Additional file 1 — ReactionExtractor. An example Java program, provided as a runnable .jar file, implementing the algorithm described in the paper. The program takes a plain text file as input and outputs all predicted reactions from the input text. Running instructions are included in the archive. The open source tools described in the paper (BANNER, OSCAR3 and OpenNLP) are all included in the archive. [file 1471-2105-13-172-S1.zip › ReactionExtractor/README.html~]

Reaction Extractor Documentation


# ReactionExtractor.jar usage instructions

The program is designed as a proof of concept and, as such, is not yet ready for proper use and is only provided for testing purposes.

The program is written is Java 6 SE. To install the program:

1. Extract this archive into a folder.
2. Open the file ‘Properties.dat’ in a text editor and change the value of ‘InChi’ to the absolute path of the file ‘cInChI-1’ within the ReactionExtractor\_lib folder.
3. Download OSCAR3 from here. Extract the archive and copy ‘oscar3-a5.jar’ to the ReactionExtractor\_lib folder. Then rename the file to OSCAR3.jar.
4. Download the BANNER gene model from here. Save it to the ReactionExtractor\_lib folder.

To use the program open a terminal/command prompt in the folder containing the ReactionExtractor.jar file and enter the following command:

`java -jar ReactionExtractor.jar -i <input file> -o <output file>`

where you should replace `<input file>` with the path to a plain text file from which reactions can be extracted and `<output file>` with the desired name of the file to which the results should be outputted — a new file will be created if it does not exist. An example input file and corresponding output file are provided in the 'Example' folder.

The program, in its current state produces this error:

```
JNI InChI has failed to load the native libraries required.

The most common problems are either the native library files being placed in
locations that JNI InChI does not know to search, or needing recompiling for
your system.

ERROR MESSAGE: Error loading InChI library: /example/directory/ReactionExtractor_lib/libinchi.so.1.01.00: /example/directory/ReactionExtractor_lib/libinchi.so.1.01.00: wrong ELF class: ELFCLASS32 (Possible cause: architecture word width mismatch)

Falling back to using non-JNI InChI - please ignore the above!
```

Don't worry about this! The program will run fine and will hopefully be fixed in a future version.

If you have any questions about the usage of the program please contact Jan Czarnecki in the Adrian Shepherd Group at Birkbeck, University of London.
