# Supplementary material for: A text-mining system for extracting metabolic reactions from full-text articles
Source: BMC Bioinformatics. 2012 Jul 23;13:172. doi: 10.1186/1471-2105-13-172 (PMC3475109; doi:10.1186/1471-2105-13-172)
Supplement: Additional file 2 — SupplementaryMaterial. An archive containing a detailed, worked example of the algorithm and the reconstructions of the tetrahydrofolate biosynthesis pathway and the fatty acid β-oxidation I pathway, together with a set of example sentences annotated with the putative entities and relationships extracted by our system. [file 1471-2105-13-172-S2.zip › README.html]

Supplementary Information


# Contents of the Supplementary Information

- ## AlgorithmExample.html

  This document contains a worked example of the reaction extraction algorithm described in the paper.
- ## Output.txt

  The extracted reactions from 74 example sentences.
- ## TetrahydrofolateBiosynthesisBioCyc.png

  An image of the tetrahydrofolate biosynthesis pathway, obtained from EcoCyc.
- ## TetrahydrofolateBiosynthesisExtracted.pdf

  A network showing the reactions predicted for the tetrahydrofolate biosynthesis pathway. Squares are small molecules, circles are enzymes, and a pair of arrows is used to denote a single reaction (the first for the interaction substrate-enzyme, and the second for the interaction enzyme-product). Items labeled green are correct; items labeled red are incorrect. The number next to a reaction indicates the number of times that reaction was extracted from the set of source texts. The reactions on the right-hand side of the figure (lying outside the blue rectangle) are reactions extracted by our algorithm that are not part of the manually-annotated tetrahydrofolate biosynthesis pathway from EcoCyc.
- ## FattyAcidBetaOxidationBioCyc.png

  An image of the fatty acid β-oxidation cycle, obtained from EcoCyc.
- ## FattyAcidBetaOxidationExtracted.pdf

  A network showing the reactions predicted for the fatty acid β-oxidation cycle. Squares are small molecules, circles are enzymes, and a pair of arrows is used to denote a single reaction (the first for the interaction substrate-enzyme, and the second for the interaction enzyme-product). Items labeled green are correct; items labeled red are incorrect. The number next to a reaction indicates the number of times that reaction was extracted from the set of source texts. The reactions on the right-hand side of the figure (lying outside the blue rectangle) are reactions extracted by our algorithm that are not part of the manually-annotated fatty acid β-oxidation cycle from EcoCyc.

  Only 2 reactions, which are contained in the blue bounding boxes, from the complete pathway shown in BioCyc were extracted successfully. There is, therefore, no dominant sequence of reactions in this reconstructed pathway.
