# Supplementary material for: A text-mining system for extracting metabolic reactions from full-text articles
Source: BMC Bioinformatics. 2012 Jul 23;13:172. doi: 10.1186/1471-2105-13-172 (PMC3475109; doi:10.1186/1471-2105-13-172)
Supplement: Additional file 2 — SupplementaryMaterial. An archive containing a detailed, worked example of the algorithm and the reconstructions of the tetrahydrofolate biosynthesis pathway and the fatty acid β-oxidation I pathway, together with a set of example sentences annotated with the putative entities and relationships extracted by our system. [file 1471-2105-13-172-S2.zip › files/AlgorithmExample.html]

A complete algorithm example


# A complete algorithm example

Consider the sentence:

> L-Arabinose isomerase catalyzes the conversion of L-arabinose to L-ribulose, the first step in the utilization of n-arabinose by Escherichia coli B/r.

1. BANNER marks up L-Arabinose isomerase as a protein.
2. OSCAR3 marks up L-arabinose, L-ribulose and n-arabinose as small molecules.
3. As only the one protein is present, L-Arabinose isomerase is assumed to be the enzyme catalysing any reactions found.
4. The following assignments are made:
   - Substrates: L-arabinose
   - Products: L-ribulose, n-arabinose
5. Reaction words are looked for between the enzyme and substrates. Conversion is found (+2 points).
6. Any keywords are searched for between substrates and products. If both reaction words and production words are found, reaction words must precede production words. Only to is found (+2 points).
7. A reaction word and a production word have been found (+2 bonus points).
8. and is looked for between last two products. Not found (0 points).
9. Catalyze (or derivatives) are looked for in the sentence. One instance found (+2 points).
10. -0.1 points for every word between substrates and products (−0.1 points).
11. -0.1 points for every word between the first and last substrate, and the first and last product (that isn't a small molecule) (-0.7 points).
12. Total points = 6.2
13. Change assignment:
    - Substrates: L-arabinose, L-ribulose
    - Products: n-arabinose
14. Score = +2 (conversion), +0 (no intervening keywords), +0 (no bonus points), +2 (catalyses), −0.1 (words between substrates), −0.7 (words between substrates and products) = 3.2
15. Change assignment:
    - Substrates: L-arabinose
    - Products: L-ribulose
16. Score = +2 (conversion), +2 (to), +2 (bonus points), +2 (catalyses), −0.1 (words between substrates and products) = 7.9
17. Change assignment:
    - Substrates: L-ribulose
    - Products: n-arabinose
18. Score = +0 (small molecule between conversion and substrate), +0 (no intervening keywords), +0 (no bonus points), +2 (catalyses), −0.7 (words between substrates and products) = 1.3
19. Change assignment
    - Substates: L-ribulose, n-arabinose
    - Products: L-arabinose
20. Score = +0 (no reaction word between protein and product), +0 (no intervening keywords, such as from), +0 (no bonus points), +2 (catalyses), −0.1 (words between products and substrates), −0.7 (words between substrates) = 1.2
21. And so on, until all combinations are made.
22. Combinations are ranked by descending score.
23. Assign the top scoring combination as a predicted reaction.
24. Move down the combinations and check against all reactions that have been assigned as a predicted reaction. If at least one small molecule in the combination is found in a predicted reaction, move to the next combination. If no small molecules are found in a predicted reaction, assign the combination as a predicted reaction. This allows the predication of multiple reactions from the same sentence.
25. Return all predicted reactions above the specified threshold — 3.0. In this case a single reaction is returned:
    - Enzyme: L-Arabinose isomerase
    - Substrate: L-arabinose
    - Product: L-ribulose
    - Score: 7.9
26. A correct prediction has been made.
