# Supplementary figures and images for: A text-mining system for extracting metabolic reactions from full-text articles
Source: BMC Bioinformatics. 2012 Jul 23;13:172. doi: 10.1186/1471-2105-13-172 (PMC3475109; doi:10.1186/1471-2105-13-172)

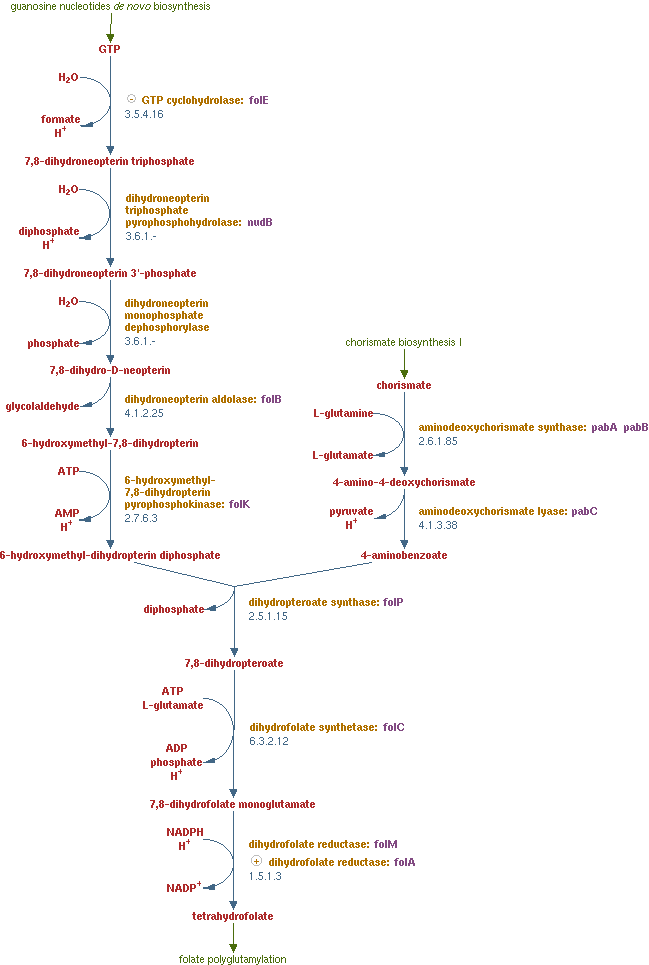

Supplement: Additional file 2 — SupplementaryMaterial. An archive containing a detailed, worked example of the algorithm and the reconstructions of the tetrahydrofolate biosynthesis pathway and the fatty acid β-oxidation I pathway, together with a set of example sentences annotated with the putative entities and relationships extracted by our system. [file 1471-2105-13-172-S2.zip › files/TetrahydrofolateBiosynthesisBioCyc.png]

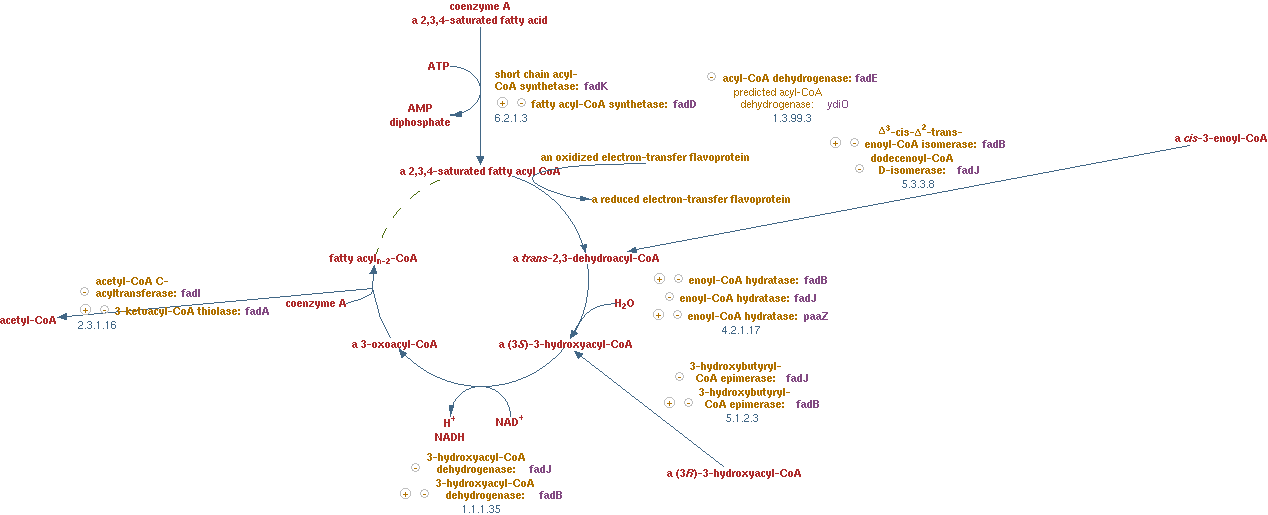

Supplement: Additional file 2 — SupplementaryMaterial. An archive containing a detailed, worked example of the algorithm and the reconstructions of the tetrahydrofolate biosynthesis pathway and the fatty acid β-oxidation I pathway, together with a set of example sentences annotated with the putative entities and relationships extracted by our system. [file 1471-2105-13-172-S2.zip › files/FattyAcidBetaOxidationBioCyc.png]

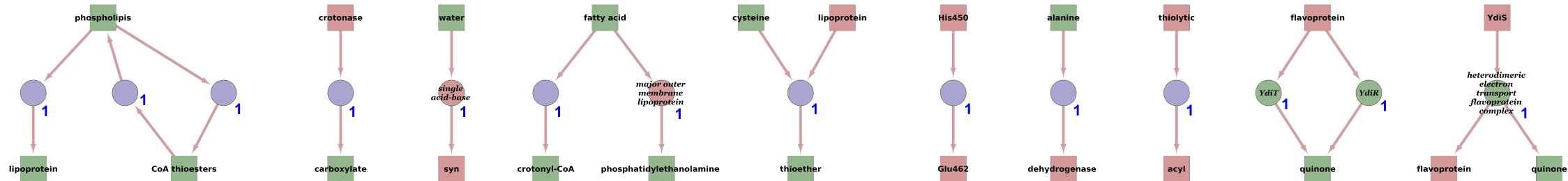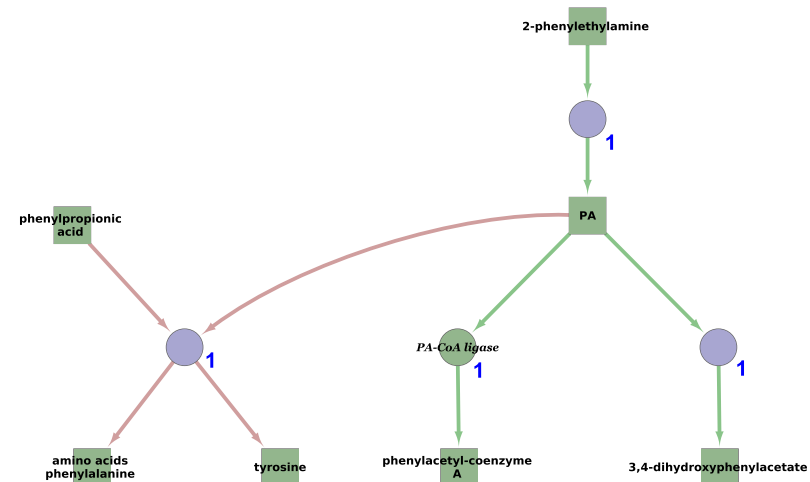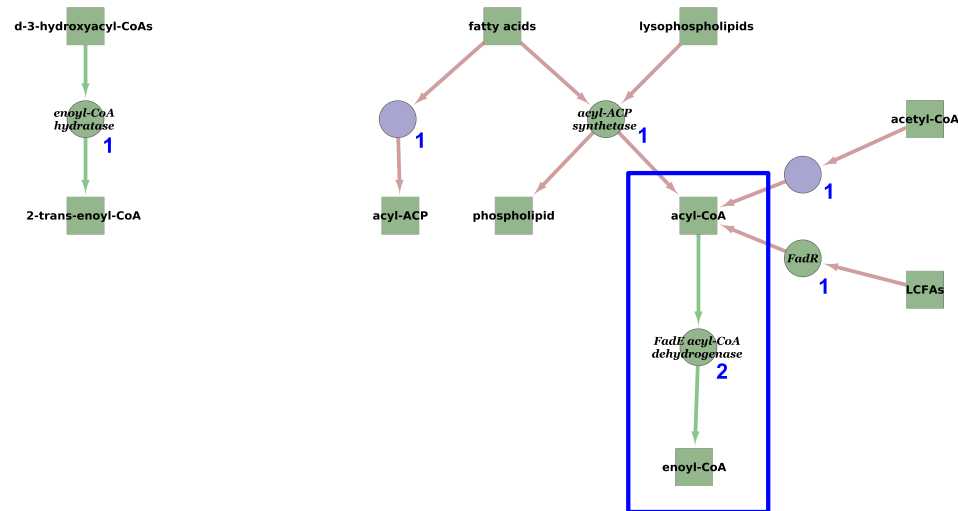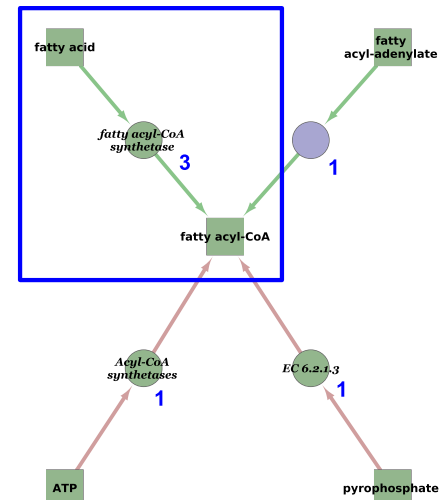

Supplement: Additional file 2 — SupplementaryMaterial. An archive containing a detailed, worked example of the algorithm and the reconstructions of the tetrahydrofolate biosynthesis pathway and the fatty acid β-oxidation I pathway, together with a set of example sentences annotated with the putative entities and relationships extracted by our system. [file 1471-2105-13-172-S2.zip › files/FattyAcidBetaOxidationExtracted.pdf]

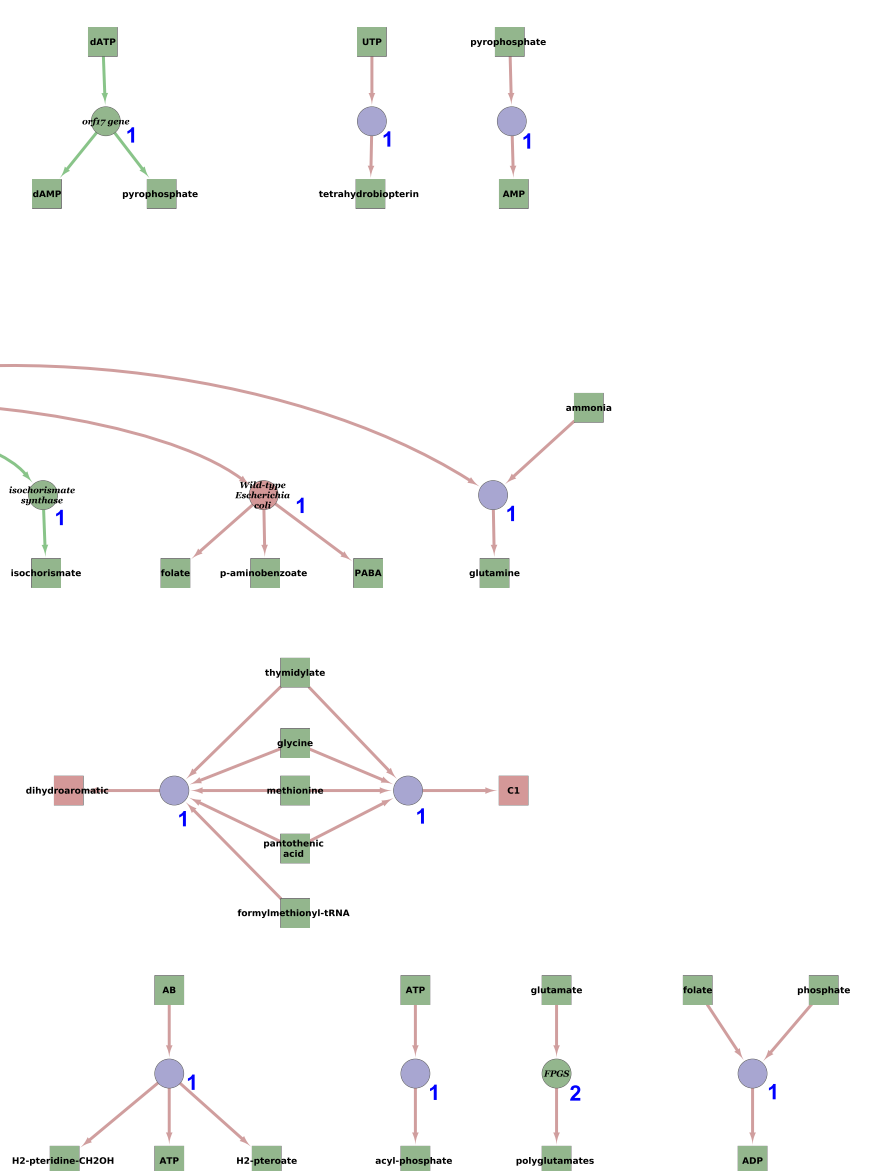

Supplement: Additional file 2 — SupplementaryMaterial. An archive containing a detailed, worked example of the algorithm and the reconstructions of the tetrahydrofolate biosynthesis pathway and the fatty acid β-oxidation I pathway, together with a set of example sentences annotated with the putative entities and relationships extracted by our system. [file 1471-2105-13-172-S2.zip › files/TetrahydrofolateBiosynthesisExtracted.pdf]
